# Computational genomics-proteomics and Phylogeny analysis of twenty one mycobacterial genomes (Tuberculosis & non Tuberculosis strains)

**DOI:** 10.1186/2042-5783-2-7

**Published:** 2012-08-28

**Authors:** Fathiah Zakham, Othmane Aouane, David Ussery, Abdelaziz Benjouad, Moulay Mustapha Ennaji

**Affiliations:** 1 Laboratoire de Virologie et Hygiène & Microbiologie, Faculté des Sciences et Techniques, BP 146, Mohammedia, 20650, Morocco; 2 Faculté des Sciences, Université Mohammed V-Agdal, Rabat, Morocco; 3 Experimental physik, Universität des Saarlandes, Postfach 151150, 66041, Saarbrücken, Germany; 4 Center for Biological Sequence Analysis, Technical University of Denmark, Lyngby, Denmark

**Keywords:** BLAST matrix, Comparative genome analysis, Evolution, *Mycobacterium tuberculosis*, Pan- core genome, Phylogeny

## Abstract

**Background:**

The genus *Mycobacterium* comprises different species, among them the most contagious and infectious bacteria. The members of the complex *Mycobacterium tuberculosis* are the most virulent microorganisms that have killed human and other mammals since millennia. Additionally, with the many different mycobacterial sequences available, there is a crucial need for the visualization and the simplification of their data. In this present study, we aim to highlight a comparative genome, proteome and phylogeny analysis between twenty-one mycobacterial (Tuberculosis and non tuberculosis) strains using a set of computational and bioinformatics tools (Pan and Core genome plotting, BLAST matrix and phylogeny analysis).

**Results:**

Considerably the result of pan and core genome Plotting demonstrated that less than 1250 *Mycobacterium* gene families are conserved across all species, and a total set of about 20,000 gene families within the *Mycobacterium* pan-genome of twenty one mycobacterial genomes.

Viewing the BLAST matrix a high similarity was found among the species of the complex *Mycobacterium tuberculosis* and less conservation is found with other slow growing pathogenic mycobacteria.

Phylogeny analysis based on both protein conservation, as well as rRNA clearly resolve known relationships between slow growing mycobacteria.

**Conclusion:**

Mycobacteria include important pathogenic species for human and animals and the *Mycobacterium tuberculosis* complex is the most cause of death of the humankind. The comparative genome analysis could provide a new insight for better controlling and preventing these diseases.

## Background

The genus *Mycobacterium* comprises more than 120 species, among them the most contagious and infectious bacteria [1]. In particular, *M. tuberculosis* (MTB) is the causal agent of tuberculosis (TB), which is an ancient microorganism infecting and killing humans for thousands of years. Several studies demonstrated that this bacterium is an intracellular microorganism restricted to mammals and its DNA is still detectable in the bones of Egyptian mummies [2-4]. It is noteworthy that the human TB could be also induced by *M. bovis*, which belongs to the MTB complex (MTBC) and principally infects cattle, but the zoonotic risk for human represents a serious problem predominantly, for those who are living at animal-human interface [5].

Moreover, according to the recent archeological studies carried out on the Siberian skeletal remains from the iron age and based on the single-nucleotide polymorphic loci PCR and the analysis of the regions of difference (RDs) of the MTBC, Taylor *et al.* confirmed the presence of *M. bovis* in those remains [6]. Before this discovery many studies suggested that the common ancestor of MTBC was *M. bovis*[7,8], but this idea has been refuted and the genetics of the MTBC explained different clues for debating in the term of their evolution over the years. The sufficient evidence of the common ancestor of these microorganisms was surrounded by a mystery and recently, the ambiguity regarding this question mark was uncovered and a new scenario was demonstrated by studying 20 variable deleted regions within the MTBC members [9]. As a result of those studies, *M.canettii* stated to be the common ancestor that did not lack those regions, unlike *M. bovis* that lost several genes are present in MTB and smooth MTB [9].

Furthermore, it has become clear that the members of MTBC were originated from a single ancestor resulted from an evolutionary bottleneck and a clonal expansion occurred 20,000 to 35,000 years ago [9-11]. In addition the progenitor of MTBC offspring was restricted in a limited geographical region (East Africa) and called “*M. prototuberculosis*” [11].

Importantly, most of the pathogenic or slow growing mycobacteria are sharing a high similarity and a strong phylogeny relationship [12,13] and interestingly, several studies confirmed that the pathogenic mycobacteria were originated from a free living progeny [9] and due to the genome reduction and the acquisition of new genes by horizontal gene transfer (HGT) [14-17] and gene rearrangement [17], their capacity of parasitism and infectiousness was developed for enabling them to cause severe and dangerous illnesses.

Indeed, the efforts worldwide focus on the combat against TB, leprosy, Buruli ulcer and other mycobacterial diseases and the medical care providers face a great challenge toward the achievement of this goal. As a result, the first sequenced mycobacterial genome was that of the reference strain *M. tuberculosis* H37 Rv [18] and it was re annotated in 2002 by Camus *et* al. [19]. In 2002, the second MTB genome sequence of the clinical strain CDC1551 was completed and a whole comparative genome analysis was done with the reference strain H37 RV based on Large Sequence Polymorphisms (LSPs) and Single Nucleotide Polymorphisms (SNPs) [20].The SNPs were also used as comparative genome markers for studying the evolution, pathogensis and molecular epidemiology of clinical MTB strains and a new phylogeny analysis based on SNPs arrangement was established by Alland *et al.*[21]. More recently, Fillol *et al.* also described the same approach and they have identified six SNP cluster groups (SCGs) and five subgroups within the MTBC members [22].

Recently, different databases were established and provided the complete genome annotation of the reference strain and other TB strains, such: TubercuList and TB database (TBDB) [23,24] and with the huge amount of mycobacterial sequences, there is a crucial need for the visualization, simplification and comparative genomics of their data for better understanding their evolutionary events and consequently, the conception of their environmental niches, mechanisms of adaptation into human and animal being, pathogencity, virulence determinants that paved the way for appropriate conditions of survival within their hosts and the development of new tools of diagnosis and drug targets for better controlling those threatening diseases [25].

Thus, a set of different approaches were used for studying the phylogeny of MTBC by fingerprinting the insertion sequence IS*6110*[26] or by the SNPs based analysis [21,22] and the phylogeny of other mycobacteial species was performed based on the extracted 16S rRNA sequences [27,28].

Recently, the availability of bioinformatics tools for genomic comparison facilitated handling, visualizing and analyzing of enormous amount of sequence information of multispecies bacterial genomes [29,30]. Therefore, this present study aims to highlight a comparative genome, proteome and phylogeny analysis by the computational and bioinformatics tools (Pan and Core genome plotting, BLAST matrix and phylogeny analysis for the comparison between twenty-one mycobacteral strains, some of them are belonging to the MTBC and other non tuberculosis mycobacterial (NTM) strains (pathogenic and free living myobacteria).

## Results

Twenty-one mycobacterial genomes were obtained from the GenBank database and used in this study, eight of them are belonging to the MTBC and thirteen genomes are representing non tuberculosis mycobacteria*.*

Table 1 summarizes a number of characteristics for each of the analyzed genome, such as size, number of predicted genes, rRNA operons, tRNA genes and GC content.

**Table 1:** Characteristics of 21 mycobacterial genomes

**Genome**	**Genome Size**	**Number of genes**	**GC content**	**5S rRNAcount**	**16S rRNA count**	**23S rRNA count**	**tRNA count**	**Gen Bank Accession Number**
Mycobacterium leprae Br4923	3268071	2720	57.8	1	1	1	45	FM211192
Mycobacterium leprae TN	3268203	2720	57.8	1	1	1	45	AL450380
Mycobacterium bovis AF2122/97	4345492	3953	65.6	1	1	1	45	BX248333
Mycobacterium bovis BCG str. Pasteur 1173P2	4371711	3988	65.6	1	1	1	47	AM408590
Mycobacterium bovis BCG str. Tokyo 172	4371711	3984	65.6	1	1	1	45	AP010918
Mycobacterium tuberculosis KZN 1435	4398250	4060	65.6	1	1	1	45	CP001658
Mycobacterium tuberculosis CDC1551	4403837	4189	65.6	1	1	1	45	AE000516
Mycobacterium tuberculosis H37Rv	4411532	3999	65.6	1	1	1	45	AL123456
Mycobacterium tuberculosis H37Ra	4419977	4034	65.6	1	1	1	45	CP000611
Mycobacterium tuberculosis F11	4424435	3950	65.6	1	1	1	45	CP000717
Mycobacterium abscessus ATCC 19977	5090491	4941	64.1	1	1	1	47	CU458896
Mycobacterium avium subsp. paratuberculosis K-10	4829781	4350	69.3	1	1	1	46	AE016958
Mycobacterium avium 104	5475491	5120	69.0	1	1	1	46	CP000479
Mycobacterium ulcerans Agy99	5805761	4241	65.4	1	1	1	45	CP000325
Mycobacterium sp. MCS	5920523	5615	68.4	2	2	2	48	CP000384
Mycobacterium gilvum PYR-GCK	5982829	5579	67.7	2	2	2	47	CP000656
Mycobacterium sp. JLS	6048425	5739	68.4	2	2	2	48	CP000580
Mycobacterium sp. KMS	6256079	5975	68.2	2	2	2	48	CP000518
Mycobacterium vanbaalenii PYR-1	6491865	5979	67.8	2	2	2	49	CP000511
Mycobacterium marinum M	6660144	5452	65.7	1	1	1	46	CP000854
Mycobacterium smegmatis str. MC2 155	6988209	6716	67.4	2	2	2	47	CP000480

The size of genomes is considerably varied between species. Moreover, the common character of mycobacterial species is their high GC content and remarkably most of the pathogenic slow growing mycobacteria have a single rRNA operon and a low number of tRNA, comparing with the RGM.

### Pan- and core- genome plot

The proteomes of the genomes against each other were predicted, extracted, and BLASTed, from all the analyzed genomes.

**Figure 1:**
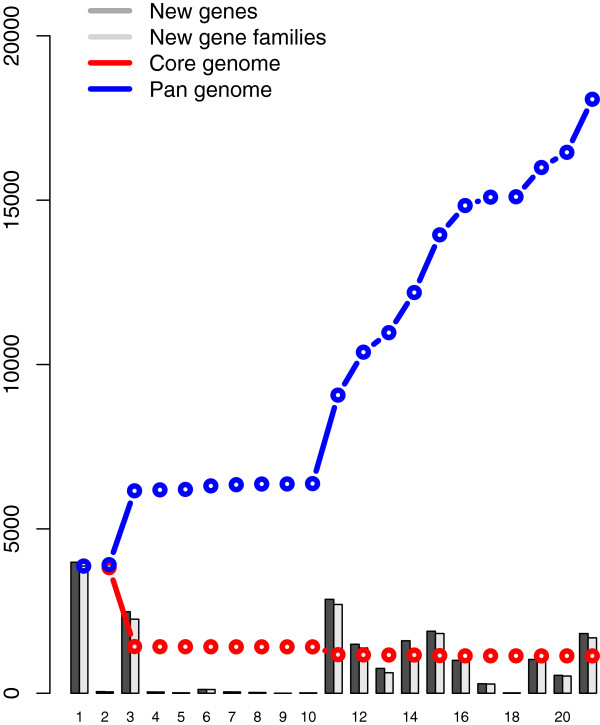
**The pan-genome (*blue line*) and core genome (*red line*) for*Mycobacterium.*** The number of discovered novel genes (*dark bars*) and novel gene families (*light-grey bars*) are also shown for each added genome.

Twenty-one Mycobacterial genomes coded for 97,304 genes in total, with 4,633 genes in average per genome were used for uncovering the pan and core genome. The pan and core-genome was calculated and the resulting plot is shown in Figure 1.

For each proteome, a BLAST search was performed against all previous proteomes and were considered conserved if they showed sequence similarity of 50% over more than 50% of the full length, as defined in the material section.

The first two genomes represented are belonging to the complex MLP and they approximately have the same pan and core genome and both of pan and core genome curves are adherent together, which indicate that most of dispensable genes are lost and the conserved genes are still persisting in those species. Since the core genome covers all genes conserved between all (sequenced) members of a species and will also contain all genes that are essential for all life forms, such as genes coding for transcription, translation, replication, and essential metabolism proteins.

As expected, moving along to the new genomes of MTBC members, there is a significant jump for both of pan and core genome curves. By the addition of the accessory genes, the pan genome is increased, in contrast of the conserved genes presented by the core genome that are dropped.

Significantly, all the species of the MTBC have the same pan and core genomes, reflecting the high degree of similarity between them.

By the addition of new genomes the variability between genomes was obviously unambiguous, especially in the pan genome, which correlates with the larger genome sizes with new accessory genes, especially in the free living mycobacteria or the RGM, that have the bigger genome sizes, as shown in the plot.

The core-genome, which represents the minimal set of conserved gene families, was dropped and at the time of calculation, based on the best fitting extrapolation; it was estimated to be approximately 1250 gene families for twenty one myocbacterial genomes.

Moreover, a total set of about 20,000 gene families within the *Mycobacterium* pan-genome were defined, including the conserved and dispensable genes.

### BLAST matrix

**Figure 2:**
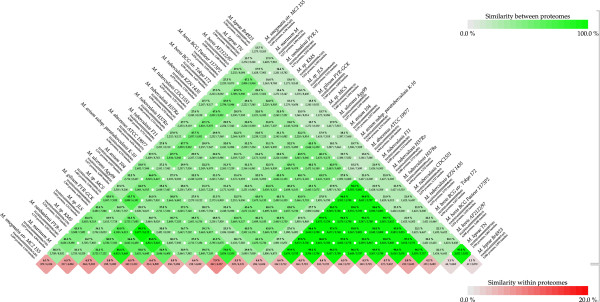
**Genomic analysis of predicted proteome for twenty one mycobacterial strains by BLAST matrix, based on pairwise whole-genome comparison.** The percentage of similarity for each combination is presented by green color between genomes and red color shows similarity within the same genome.

The results of the genomic analysis of predicted proteome are visualized by BLAST matrix Figure 2, in which the pairwise whole-genome comparison was done.

The percentage of similarity for each combination is presented by green color between genomes and red color shows similarity within the same genome.

The comparison of the MTB the species reveals a high similarity among them, ranging between; 96.1–99.2% (colored with darker green, an indicative of a higher degree of similarity within these strains).

The similarity between *M. bovis* with MTB strains was ranged between; 93.7–94.8% and between *M.bovis* and attenuated *M. bovis* BCG vaccine strains were 96.7% and 97.1%, with *M bovis BCG str. Pasteur 1173P2, M bovis BCG str. Tokyo 172* respectively.

The similarity between the members of the complex MLP was of 97.5% and it was very low between the members of MTBC and MLP. Interestingly, the similarity between the MTBC and *M.Marinum* was ranged between 47.0–47.7% and less similarities were found between the MTBC, *M ulcerans Agy99* and the MAV complex.

Furthermore, the similarity between pathogenic and free living mycobacteria was very low, presented by the pale green color between their predicted proteomes.

### Phylogentic analysis

For studying the evolution of the mycobacterial species a phylogenetic tree was constructed, based on the extracted 16S rRNA sequences.

**Figure 3:**
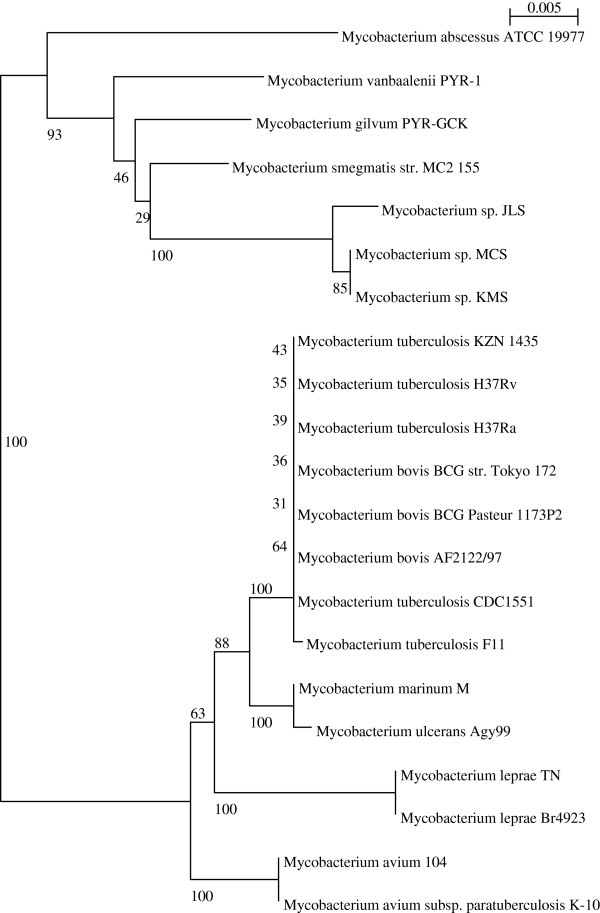
**Phylogenetic tree based on extracted 16S rRNA sequences of twenty one mycobacterial species.** The members of the complex *Mycobacterium tuberculosis* are clustered together and sorted from the same clade.

The results of Phylogentic analysis are shown in Figure 3; remarkably the free living mycobacteria are separated by a complete outlier (in exception of *M. abscessus*) and the pathogenic mycobacteria represent the other outlier, the later is composed of four separated clades: MTBC, *M. ulcerans-marinum*, MAV and MPL complex. The horizontal line at the top (in this case, 0.005) is used to provide a rough measure of genetic distance. Moreover, the booststraps values are also indicated on the constructed phylogenetic tree Figure 3.

Significantly, the members of MTBC are clustered together; demonstrating a high similarity between them and confirming that the MTBC members are sharing the same ancestor in their evolutionary events and there is a high relatedness between them and other pathogenic NTM; especially *M. Marinum,* as mentioned above in the results of the BLAST matrix.

## Discussion

The genus *Mycobacterium* comprises more than 120 species; among them the saprophytes that adopted for free living lifestyle. Others are contagious, infectious and cause dangerous diseases for human and animals [1]. Moreover, the availability of complete genome sequences of these important pathogens provided a wealth of information that could enable understanding the mechanisms of evolution, pathogenesis and systematically potential targets of drug discovery [25]. In general, the mycobacteria belong to the phylum of Actinobacteria that are characterized by their large genome size and high GC content [30] and owing to their different lifestyles and environmental niches, the sizes of their genomes are varied [29].

Remarkably, the pathogenic slow growing mycobacteria had undergone to genome reduction [29] and consequently, they have a single rRNA operon and a low number of tRNA, comparing with the RGM [30]. In this context, the loss of genes played an important role in the evolution of slow growing mycobacterial pathogens [31] and some of those genomes were suffered of an extensive genome downsizing like the MLP complex [32]. On otherwise the members of MTBC were subjected to moderate genome reduction and concomitantly, acquired new genes towards the speciation to the parasitic life style in mammalian macrophages.

With the considerable genome size differences among the species included in this study, the number of added new genes per genome has increased the pan genome and the discovery of new genome sequences could add new genes to the current pan genome.

Unsurprisingly, the results of the core genome are in correlation with a previous experimental study realized by Marmiesse *et al*., in which they have estimated about 1439 genes as minimal set of conserved genes in the core genome of MTB and MLP, among them 219 genes that code for proteins show no similarity with proteins from other mycobacterial species [33]. Consequently, this approach could help in the designation of new TB vaccines as reported by Jungblut *et al.*[34].

In reality, one genome sequence is not enough for the development of a valuable vaccine and the definition of pan and core genome can provide a new insight for attaining this goal.

Even more, the comparative genomic analysis of predicted proteome by BLAST matrix showed a high similarity among MTB strains as already reported [35]. The similarity values between MTB strains, *M.bovis* and attenuated *M. bovis* BCG vaccine strains were reasonable and the slight difference between the two vaccine strains (96.7%, 97.1%) could be attributed to the lack of RD 14 and the restricted duplication DU1 in the BCG *Pasteur* strain [36]. Interestingly, the similarity between the MTBC and *M.marinum* was ranged between 47.0–47.7%. Regardless of the bigger genome size of *M. Marinum*, the close relatedness between those two species was documented and 3000 orthologs were shared between both genomes [37]. This support the hypothesis of the decendy of MTB from an environmental *Mycobacterium* and due to the genome downsizing and the acquisition of new genes by HGT [14,38], their capability of parasitism was evolved towards the mammalian macrophages [14,37].

*M ulcerans Agy99,* which evolved from the same ancestor of *M. marinum* and has shown a less similarity with MTBC members (43%) and notably, this bacterium suffered of deletions, mainly, in the ESX1 locus [39], which is present in MTB and *M marinum*.

Significantly, *M. avium*s sp. *Paratuberculosis* strain K-10 with more than 3,000 homologous genes with MTB [40] showed a less similarity and the least similarity was found with the complex MLP and this could be attributed to the loss of PE_PGRS proteins in both of those species [41], Furthermore, the absence of many genes in MAV and the presence of pseudogenes in MLP elucidate the specificity of their virulence, tropism, the ability of cultivation and drug susceptibility pattern [42].

For better understanding the evolution of mycobacterial strains a phylogenetic tree was constructed based on 16 rRNA sequences, which allows the identification of most species within the genus *Mycobacterium* and can separate between the slow and RGM [27] and can be used for the systematic phylogeny analysis [28].

Unsurprisingly, the results of phylogeny analysis were in correlation with the previous results of genomic analysis of predicted proteome, which leads to an assertion that the ancestral MTBC and *M. marinum* genomes might be descended from the same ancestry owing to their close genetic relationship and phylogenetic relatedness.

Markedly, before the evolutionary bottleneck and clonal expansion of MTB members, the ancestral *M. prototuberculosis* species had acquired the particular Rv0986-8 virulence operon by HGT from an alpha proteobacterium [15] and evidently this operon still exists only in MTBC members.

Moreover, our study confirmed the high phylogenetical relationship among: MTBC, *M.marinum- ulcerans*, MAV and MLP complex, showing that the pathogenic slow growing mycobacteria define a distinct and common line of evolutionary descent from a free living bacterium.

Interestingly, the RGM have also acquired different genes toward the speciation to their environmental niches, like the acquisition of PAH Catabolism Genes in: *M sp.MCS, M gilvum PYR-GCK, M sp. JLS, M sp KMS* and *M vanbaalenii PYR-1*) [43], which enabled them to degrade polycyclic aromatic hydrocarbons. Significantly, many of RGM are ubiquitous in the environment and could become pathogenic for humans and induce opportunistic incurable infections like, *M. abscessus* and *M. smegmatis* due to their resistance against bactericidal agents.

Simultaneously, those RGM still share some conserved genes with the slow growing pathogenic mycobacteria, like the locus ESX3 which is highly conserved among all mycobacterial species [37] and as a result of comparative genomics this locus was exploited as a recombinant *M. smegmatis* vaccine against MTB [44].

Thus, the combination of different sets of computational genome, proteome and phylogeny analysis could easily visualize the evolutionary events and similarity relationships between mycobacterial species, which could help for better improving new diagnosis approaches and vaccines against TB and other mycobacterial diseases.

## Conclusion

Mycobacteria include important pathogenic species for humans and animals, and the *Mycobacterium tuberculosis* complex can be a major cause of death in humans. With the progress of molecular diagnosis of infectious diseases in this era of huge amounts of DNA sequences, the visualization of data becomes one of the most important priorities to facilitate the analysis and the interpretation of the evolutionary events of the bacteria.

Furthermore, the combination of different sets of bioinformatics tools could offer a good comparative genome analysis, which can provide new insights for better controlling and preventing infectious diseases.

## Materials & methods

### Bacterial strains

The main source of information is: http://www.ncbi.nlm.nih.gov/ GenBank at NCBI, from which twenty one genomes of pathogenic and free living mycobacterial strains were retrieved for this study. Among them eight strains belong to the MTBC: the reference strain *M tuberculosis H37Rv*, the attenuated laboratory strain *M tuberculosis H37Ra*, the modern laboratory strains: *M tuberculosis KZN 1435, M tuberculosis CDC1551, M tuberculosis F11,* the bovine TB strain *M bovis AF2122/97* and two attenuated vaccine strains *M bovis BCG str. Pasteur 1173P2, M bovis BCG str. Tokyo 172*.

Two genomes belong to the complex of *M. Leprae* (MLP), which is responsible of leprosy in human: *M leprae Br4923, M leprae TN*.

Other two strains were among the complex of *M. Avium* (MAV) that induce Johne’s disease in cattle and other ruminants: *M avium subsp. paratuberculosis K-10, M avium104*. The causative agent of Buruli ulcer *M ulcerans Agy99* and *M marinum*that causes granulomatous lesions in fish and sometimes skin lesions in human were also included.

*M abscessus ATCC 19977* that induces cystic fibrosis and severe lung disease was the sole pathogen in the rapid growing mycobacteria (RGM), the rest were among the free living mycobacteria: *M sp.MCS, M gilvum PYR-GCK, M sp. JLS, M sp KMS* and *M vanbaalenii PYR-1* and *M. smegmatis str. MC2155*.

The features of those genomes (Genome length, GC content, number of genes, tRNA genes and rRNA operons) are summarized in (Table 1).

### Computational tools

The calculations of the BLAST Matrix, Pan- Core genome plot and the prediction of 16S ribosomal RNA using RNAmmer were performed using in-house scripts on computers at the Centre of Biological Sequence Analysis, in Denmark.

1. Pan- and Core- genome plot construction

Pan- and core-genome plots are graphs that display to what extent gene families are conserved within a set of genomes. The method used here is an approximation as described previously for calculating the pan- and core-genome of 32 *E. coli* genomes [45].

Conservation is evaluated by first blasting the predicted proteomes of the genomes against each other. For each proteome, a BLAST search is performed against all previous proteomes. The result is a set of numbers specific for that time point that represents the proteome in the order of the input list, showing:

Two genes are considered to belong to the same gene family if the two are more than 50% identical over more than 50% of their length (Figure 1).

· Number of new genes

· Number of new families

· Size of core genome

· Size of pan genome

2. BLAST matrix construction from hypothetical genes/proteins

A BLAST matrix is a comparison of proteomes (proteins from a genome) used to estimate how many proteins is found in common between two genomes.

All annotated proteins of all 21genome sequences currently available have been collected, and blasted each of the individual sequences against the collection. For each bacterium, the number of genes distinct for that organism and the number of genes shared with the other species have been extracted. The BLAST matrix was constructed, showing protein similarity between all combinations of mycobacterial genomes (Figure 2), and reflect to some extent an evolutionary distance or similarity between the individual species.

The algorithm of the BLAST matrix is simple, and consists of three parts:

I. A match requirement.

When two proteins are blasted against each other, the matching region should have at least 50% identity, while covering at least 50% of the length of the longer protein.

II. A protein family building scheme.

The protein families were built by blasting proteins together. If they satisfy the match criteria, they are clustered together. If one of the proteins in the match is a member of an existing gene family, the other protein is added to the same family.

III. Comparisons between and within proteome.

The protein families were created both within and between proteomes. For comparisons building between proteome, every protein within one proteome is compared to all proteins in the other proteome.

3. 16 Ribosomal RNA phlogenetic tree construction

The sequences encoding 16S ribosomal RNA were predicted using RNAmmer [46] and were extracted for the set of twenty-one genomes. Alignment was done with ClustalX.

A phylogenetic tree was constructed using Bootstrap neighbour-joining method and visualized by NJPlot (Figure 3).

## Abbreviations

MTB: *Mycobacterium tuberculosis* ; MTBC: MTB complex; TB: Tuberculosis; NTM: Non Tuberculosis Myocbacteria.

## Competing interests

The authors declare that they have no competing interests.

## Authors’ contributions

FZ performed analyzed the data and wrote the first draft of the manuscript. AO performed the computational analysis and participated in the design of the study. DU made substantial contribution to conception and design of the study and participated in data interpretation. All authors read and approved the final manuscript.
